# Understanding Splenomegaly in Myelofibrosis: Association with Molecular Pathogenesis

**DOI:** 10.3390/ijms19030898

**Published:** 2018-03-18

**Authors:** Moo-Kon Song, Byeong-Bae Park, Ji-Eun Uhm

**Affiliations:** 1 Department of Hematology-Oncology, Hanyang University Hanmaeum Changwon Hospital, Changwon 51497, Korea; song9676@hanmail.net; 2 Division of Hematology-Oncology, Department of Internal Medicine, Hanyang University College of Medicine, Hanyang University Hospital, 222-1, Seongdong-Gu Wangsimni-Ro, Seoul 04763, Korea; mksong9676@gmail.com

**Keywords:** splenomegaly, extramedullary hematopoiesis, myelofibrosis

## Abstract

Myelofibrosis (MF) is a clinical manifestation of chronic BCR-ABL1-negative chronic myeloproliferative neoplasms. Splenomegaly is one of the major clinical manifestations of MF and is directly linked to splenic extramedullary hematopoiesis (EMH). EMH is associated with abnormal trafficking patterns of clonal hematopoietic cells due to the dysregulated bone marrow (BM) microenvironment leading to progressive splenomegaly. Several recent data have emphasized the role of several cytokines for splenic EMH. Alteration of CXCL12/CXCR4 pathway could also lead to splenic EMH by migrated clonal hematopoietic cells from BM to the spleen. Moreover, low Gata1 expression was found to be significantly associated with the EMH. Several gene mutations were found to be associated with significant splenomegaly in MF. In recent data, *JAK2* *V617F* homozygous mutation was associated with a larger spleen size. In other data, *CALR* mutations in MF were signigicantly associated with longer larger splenomegaly-free survivals than others. In addition, MF patients with ≥1 mutations in AZXL1, EZH1 or IDH1/2 had significantly low spleen reduction response in ruxolitinib treatment. Developments of JAK inhibitors, such as ruxolitinib, pacritinib, momelotinib, and febratinib enabled the effective management in MF patients. Especially, significant spleen reduction responses of the drugs were demonstrated in several randomized clinical studies, although those could not eradicate allele burdens of MF.

## 1. Introduction

Myelofibrosis (MF) is a clinical manifestation of chronic BCR-ABL1-negative chronic myeloproliferative neoplasms (MPNs), which is similar to essential thrombocythemia (ET) and polycythemia vera (PV), characterized by progressive bone marrow (BM) fibrosis, extramedullary hematopoiesis (EMH), progressive splenomegaly, and leukoerythroblastosis in the blood [1]. It includes primary myelofibrosis (PMF) that is arising from a previous ET or PV (post-ET or post-PV). In the case of MF, there is clonal proliferation of pluripotent hematopoietic stem cells (HSCs), where the cell population releases several cytokines and growth factors in the BM, leading to marrow fibrosis, stromal changes, and EMH [2,3].

The Janus kinase 2 (*JAK2*) V617F mutation is present in 60% of the patients with PMF or post-ET MF and 95% of those with post-PV MF [4,5]. The mutation now occupies an important position in the understanding of the molecular pathogenesis of MF. In addition, mutations in the thrombopoietin receptor gene (*MPL*) have been found in 10–25% of patients with PMF and post-ET MF [6], whereas mutations in the calreticulin gene (*CALR*) have been observed in *JAK2-* and *MPL*-negative patients with PMF (75%) and post-ET MF (50–60%) [7].

Splenomegaly is one of the major clinical manifestations of MF and is directly linked to splenic EMH. The emergence of EMH is associated with the abnormal trafficking patterns of clonal hematopoietic progenitor cells (HPCs) and HSCs due to the dysregulation of the BM microenvironment. Progressive splenomegaly is significantly associated with debilitating symptoms, such as early satiety, deteriorative abdominal pain, portal hypertension, decreased physical activity, and the progression of cytopenia due to splenic sequestration in MF [8]. Because the median age of patients with MF is around 65 years at diagnosis, disease-associated complications are often compounded by concurring medical conditions, such as diabetes, hypertension, atherosclerotic, and obesity, all of them being linked to Metabolic Syndrome, and thus non-alcoholic fatty liver disease [9]. These conditions are ultimately associated with liver-spleen axis, the accounted role of chronic low-grade inflammation, and insulin resistance [10].

The symptoms of splenomegaly are associated with spleen size. In one study, palpable splenomegaly was observed in 80% of the asymptomatic patients, and about 10% of the patients with MF showed severe symptomatic splenomegaly when diagnosed with MF [11].

## 2. Somatic Mutations Associated with the Pathogenesis of MF

The JAK tyrosine kinase family consists of JAK1, JAK2, JAK3, and tyrosine kinase 2 (TYK2), which is associated with the cytoplasmic juxtamembrane region of cytokine receptors, including the MPL, erythropoietin receptor (EPOR), and granulocyte colony-stimulating factor receptor (GCSF-R) [12]. Activation of the receptors by cytokines, such as thrombopoietin (TPO), erythropoietin, and GCSF, leads to dimerization and recruitment of JAKs. Then, the JAKs phosphorylate the key tyrosine residues in the receptors and activate the downstream signal transducer and activator of transcription (STAT) pathway, leading to an alteration in the transcription and expression of the genes that control cell proliferation and survival [13].

The *JAK2* V617F mutation is present in exon 14 on chromosome 9p24. When JAK2-V617F binds to cytokine receptors, such as MPL, EPOR, and GCSF-R, restricted signaling occurs via the STAT 3/5, phosphatidylinositol-3-kinase (PI3K)/AKT, and the RAS/mitogen-activated protein kinase (MAPK) pathways, resulting in the augmentation in gene expression and increase in all three myeloid lineages [4].

*MPL* is a thrombopoietin receptor and key to the growth and survival of megakaryocytes [6]. Somatic mutations in *MPL* (commonly, *MPL* W515K, and *MPL* W515L) cause its spontaneous activation, leading to cytokine-independent activation of the downstream JAK-STAT pathway. *MPL* W515K is involved in stem cell-derived events with both myeloid and lymphoid progenitors [14].

Calreticulin (CALR) is a multifunctional calcium-binding protein and not a signaling molecule; it is located primarily in the endoplasmic reticulum [7]. In recent studies, *CALR* mutants were found to activate MPL and the downstream *JAK-STAT* signaling pathway, as *CALR* mutants are abnormal chaperones and traffic with MPL to the cell surface [7,15].

Overall, somatic mutations of *JAK2*, *MPL*, and *CALR* are now recognized as driver mutations responsible for the MPN phenotype. Moreover, other discovered subclonal mutations in *ASXL1*, *EZH2*, *CBL*, *IDH1/2*, *TP53*, and *SRSF2* are now known to be associated with disease progression in MF [16,17].

## 3. Correlation between EMH in the Spleen and Molecular Pathogenesis in MF

MF is characterized by abnormal trafficking of HSCs and hematopoietic progenitor cells (HPCs), leading to their migration from the BM and the engraftment to EMH sites [18]. Expansion of hematopoietic space, such as the spleen, outside of the BM is usually observed in MF. Recently, it was suggested that HSCs and HPCs migrate from the BM to the splenic microenvironment in MF, leading to continuous proliferation of malignant clones and progressive splenomegaly. Several recent data have emphasized the role of several cytokines that are associated with EMH. Stem cell factor (SCF) in mouse model was highly expressed by endothelial cells and Tcf21+ stromal cells in red pulp od spleen, leading to splenic EMH [19]. In other data, JAK2 V617F cells and spleen size expanded much more robustly in the presence of tumor necrotic factor-α (TNF-α) [20]. Moreover, fibrogenic cytokines, such as platelet-derived growth factor (PDGF), transforming growth factor-β (TGF-β), and basic fibroblast growth factor (bFGF) were involved in pathogenesis of MF and splenomegaly [21]. Intramedullary accumulation of platelet factor 4 (PF4) and high level of interleukin-8 (IL-8) in MF were also suggested to promote EMH in liver and spleen [22,23].

The C-X-C motif chemokine ligand 12 (CXCL12) is produced by mesenchymal stromal cells and osteoblasts in the BM and is known to play a critical role in the maintenance and development of HSCs in the BM [24,25]. Recently, Miwa et al. demonstrated that CXCL12 is also produced by sinus endothelial cells of the red pulp in EMH-positive spleens [26]. CXCL12 has also been found to play a crucial role in the migration and maintenance of HSCs in EMH.

CXCL12 binds to the G-protein-coupled receptor, C-X-C chemokine receptor type 4 (CXCR4), on hematopoietic cells and other cells [25]. Then, CXCL12/CXCR4 signaling stimulates hematopoiesis in the BM. Particularly, hypoxic conditions due to insufficient hematopoiesis in the BM, as in the case of MF, can alter the CXCL12/CXCR4 axis, resulting in the migrating of HSCs and HPCs from the BM to the spleen [26]. Moreover, the alteration could stimulate and maturate the HSCs in the spleen, thus resulting in EMH [27]. In several studies, alterations in the CXCL12/CXCR4 axis, including the abnormal processing of CXCL12 in a pathological environment and the decreased expression of CXCR4 in MF have been identified [27,28,29,30].

**Figure 1:**
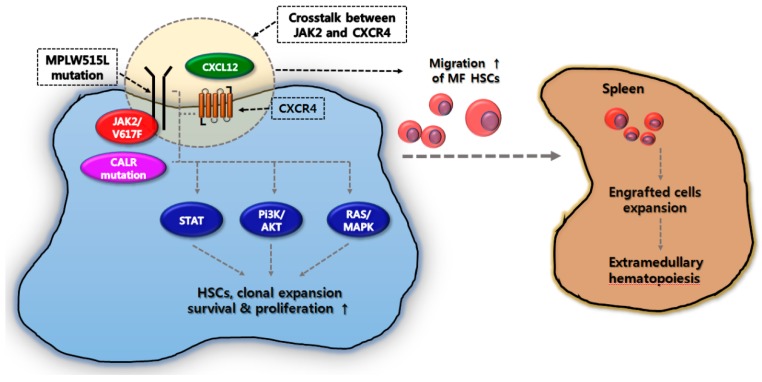
Splenic extramedullary hematopoiesis in myelofibrosis. Cooperation between *JAK2* signaling and C-X-C motif chemokine ligand 12 (CXCL12)/C-X-C chemokine receptor type 4 (CXCR4) axis activates downstream signal transducer and activator of transcription (STAT), phosphatidylinositol-3-kinase (PI3K/AKT), and RAS/MAPK pathways, leading to clonal expansion of hematopoietic stem cells (HSCs) and hematopoietic progenitor cells (HPCs) in the bone marrow. Moreover, it encourages the transfer and engraftment of the HSCs and HPCs to the spleen.

A previous study demonstrated that *JAK2* is involved in CXCL12/CXCR4-mediated cell transfer and engraftment [31]. Recently, Abdelouahab et al. demonstrated that *JAK2* activates MPL-mutant MO7e cells that promote CXCR4 signaling [32]. In the data, the crosstalk between oncogenic *JAK2* activation and CXCL12/CXCR4 signaling increased CXCL12-dependent migration and the downstream activation of the STAT, PI3K/AKT, and RAS/MAPK pathways (Figure 1). Moreover, *JAK2* inhibition by ruxolitinib or AZD1480 (*JAK1/JAK2* inhibitor) reversed the enhanced migration response. This data demonstrate that oncogenic JAK2 activation as a driver mutation spontaneously activates the CXCL12/CXCR4 pathway and encourage EMH, resulting in progressive splenomegaly. 

In addition, Gata1 is a member of the GATA family of transcription factors that are indispensable for the appropriate maturation of hematopoietic cells of many lineages, including erythroid cells and megakaryocytes [33]. MF is associated with the reduced expression of Gata1, which is a phenotypic modifier, in megakaryocyte [34]. Recently, Zingariello et al. demonstrated that in the mouse model, Gata1 deficiency was associated with a hyperactive TPO/MPL axis [35]. Gilles et al. evaluated the effect of MPLW515L mutation on *GATA1* expression in a different megakaryocyte cell line [36]. In the data, the MPLW515L mutant cells expressed significantly low levels of Gata1 than the parental cells. This means that the activation of JAK-STAT pathway signaling leads to downregulation of Gata1 in the megakaryocytes. Moreover, low Gata1 expression was found to be significantly associated with EMH, independent of CXCR4 pathway activation [37]. Therefore, each mechanism, namely the CXCL12/CXCR4 pathway and low Gata1 expression, individually leads to the progression from EMH to splenomegaly in MF patients.

## 4. Gene Mutations Associated with Splenomegaly in MF

The *JAK2* V617F mutation has been associated with high blood cell counts, history of thrombosis or pruritus, and the low requirement of transfusion for follow-up duration. Barosi et al. investigated the usefulness of the *JAK2* V617F mutation in explaining the phenotype variations [38]. In the data, PMF patients with the *JAK2* V617F homozygous mutation showed higher proliferative profiles, such as larger spleen size (*p* = 0.003) and higher white blood cell count (*p* = 0.003) than those with wild-type or heterogeneous mutations (Table 1).

Rumi et al. evaluated the clinical impacts of the driver mutations of *JAK2*, *CALR*, and *MPL* in patients with PMF [39]. In the data on large splenomegaly, which is defined as enlargement >10 cm from the left costal margin, *CALR* mutation-positive patients with MF had a significantly longer period of large splenomegaly-free survival than mutation-negative patients with MF (*p* < 0.001). 

Recently, Patel et al. investigated whether genetic profiles impact the clinical outcomes for patients with MF (PMF, post-ET MF, and post-PV MF) that are treated with ruxolitinib [40]. In their study, spleen response was not associated with *JAK2*, *CALR*, *MPL*, or triple-negative mutation status. However, patients with ≥1 mutation in *ASXL1*, *EZH1*, or *IDH1/2* and those with ≥3 mutations of any type were significantly less likely to have a spleen response than those with no mutations in *ASXL1*, *EZH1*, or *IDH1/2* (*p* = 0.01), and those with ≤2 mutations (*p* = 0.001).

## 5. Reduction in Splenomegaly as a Benefit of *JAK* Inhibitors in MF

The discovery of *JAK2* V617F mutation has led to the development of *JAK* inhibitors for the management of MF. Ruxolitinib is the first FDA-approved selective *JAK1*/*JAK2* inhibitor and has moderate activity against *TYK2* and *JAK3*. In the phase 1/2 trial, it was well tolerated, with thrombocytopenia as the dose-limiting toxicity [41]. Clinical approval of ruxolitinib for patients with intermediate and high-risk MF was based on the results of the phase 3 randomized COMPORT-I and -II trials that demonstrated a significant reduction in splenomegaly in the ruxolitinib group over the best available therapy (BAT) or placebo group (Table 2) [42,43]. In the COMPORT-I study, ≥35% spleen volume reduction (SVR) in computed tomography (CT)/magnetic resonance imaging (MRI) at 24 weeks of treatment was 41.9% in the ruxolitinib group versus 0.7% in the placebo or the best available therapy (BAT) group (*p* < 0.001; Table 2) [42]. Meanwhile, the COMPORT-II study showed that the SVR at 48 weeks was 28.5% in the ruxolitinib group versus 0% in the BAT group (*p* < 0.001) [43].

Pacritinib is an oral selective *JAK2* (wild and mutant type) inhibitor and also targets fms-like tyrosine kinase 3 (FLT3), colony-stimulating factor 1 receptor, and inerleukin-1 receptor-associated kinase 1 without meaningful inhibition of *JAK1*. It has an advantage as a relatively non-myelosuppressive *JAK2* inhibitor [44]. In two randomized phase-3 trials with pacritinib therapy, significant reduction in spleen size was demonstrated. The PERSIST-1 trial compared the efficacy of pacritinib (400 mg/day, *n* = 220) and BAT (*n* = 107) in MF patients. At 24 weeks of the study, SVR of ≥35% on MRI was achieved in 19% of the pacritinib group versus 5% of the BAT group (*p* = 0.0003, Table 2) [45]. The PERSIST-2, randomized phase-3 1:1:1 clinical trial compared two different dose groups of pacritinib (400 mg/day, *n* = 104; 200 mg bid, *n* = 107) and BAT (*n* = 100) involving MF patients. At 24 weeks, the SVR of ≥35% in the pooled pacritinib group was 18%, but in the BAT group, it was only 3% (*p* = 0.001) [46]. In the two randomized clinical trials, MF patients in the pacritinib arm showed a greater reduction in splenomegaly, total symptom score, and transfusion requirement than those in the BAT arm (Table 1).

Momelotinib is a potent and selective *JAK1*/*JAK2* inhibitor with the feature of alleviating anemia due to reduced production of hepcidin in the liver. SMPLIFY-I, a phase-3 head-to-head clinical trial of momelotinib versus ruxolitinib involving *JAK* inhibitor-naïve MF patients, demonstrated non-inferiority in SVR response at 24 weeks (26.9% in the momelotinib group versus 29% in the ruxolitinib group (*p* = 0.011) [47]. In SIMPLITY-II, the second phase 3 trial, MF patients that were previously exposed to ruxolitinib were randomized to momelotinib therapy versus BAT [48]. In this study, the momelotinib group did not achieve superior SVR response at 24 weeks as compared to the BAT group (*p* = 0.90; Table 2).

Fedratinib is a selective and potent *JAK2* inhibitor, targets FLT3, and is a rearranged during transfection (RET) kinase [49]. It inhibits the growth of erythroid colonies in the presence of *JAK2* V617F, *MPL* W515K, and *JAK2* exon 12 mutations and is more selective for *JAK2* than for *JAK1* and *JAK3*. In phase 3 of the randomized JAKARTA trial, two dose groups of fedratinib (400 mg once daily, *n* = 96; 500 mg once daily, *n* = 97) were compared with the placebo group (*n* = 96) [50]. At 28 weeks, 36% and 40% of patients treated with 400 mg and 500 mg of fedratinib, respectively, achieved an SVR response of ≥35%, while only 1% of the placebo group achieved the SVR response (*p* < 0.0001; Table 2). However, the trial was suspended because of the emergence of significant neurotoxicity and Wernicke encephalopathy in several cases.

Although reduction of increased spleen size could be possible by development of the JAK inhibitors, non-responders remain present. For the patients with refractory symptomatic splenomegaly and/or portal hypertension, splenectomy might be a treatment option, but should only be considered if the qualifying patient has an adequate life expectancy [51]. Similarly, palliative splenic irradiation may be indicated for patients with highly symptomatic splenomegaly and adequate platelet count [52].

## 6. Conclusions

Splenomegaly is a cardinal feature and is associated with splenic EMH in MF. In previous studies, several cytokines, such as SCF, TNF-α, PDGF, TGF-β, bFGF, PF4, and IL-8 contributed to the expansion of malignant clones from the BM to the spleen as an EMH site, leading to the development of splenomegaly in MF patients. Recently, *JAK2* activation in MPL-mutant cells was found to support the CXCL12/CXCR4 pathway and activate downstream JAK-STAT, PI3K/AKT, and RAS/MAPK activation pathways, leading to the expansion of malignant clones, EMH, and splenomegaly in MF patients [32]. Moreover, low Gata1 expression is thought to transfer and engraft from the BM to the EMH site in the spleen [34,35,36,37]. Ultimately, these mechanisms linked to EMH result in an enlarged spleen in MF. 

The understanding of driver mutations has preceded the development of *JAK* inhibitors for the treatment of MF. Several *JAK2* inhibitors have been found to effectively reduce spleen volume in MF in several randomized clinical trials [42,43,44,45,46,47,48,50]. *JAK2* mutant HPCs derived in the spleen are supposed to be sensitive to *JAK* inhibition. However, *JAK2* inhibitors did not eradicate *JAK2* V617F allele burden because MF HSCs survive. Recent data demonstrated that the eradication of *JAK2* V617F allele burden by *JAK2* inhibition is not successful because several coexisting additional genetic mutations, such as *TET2*, *ASXL1*, and *IDH1/2* mutations might participate in clonal expansion of BM HSCs [53].

Despite the clinical relevance, increased spleen size has not been proven as a significant prognostic factor in major prognostic models, including International Prognostic Scoring System (IPSS), Dynamic IPSS, Dynamic IPSS plus, MF Secondary to PV, and ET-Prognostic Model and Mutation-Enhanced IPSS 70 in patients with MF [11,54,55,56,57]. However, it is doubtful whether the data is completely reliable in MF because the assessment of spleen size in several studies is only dependent on physical examination. In some clinical data, larger splenomegaly was linked to poor prognosis in patients with MF [58,59]. Moreover, our recent study showed that large spleen volume by CT at diagnosis could predict poor survival in PMF, despite the small study sample and retrospective design [60]. In a recent data, splenomegaly larger than 10 cm was also a significant pre-treatment factor negatively correlating response of ruxolitinib treatment in MF [61]. In this regard, we believe that a well-designed prospective study with volumetric assessment of spleen at diagnosis of MF could better predict the prognosis

Splenomegaly is associated with several clinical symptoms, genetic mutations, prognostic factors, and EMH in MF. However, the clinical value of the spleen appears to be underestimated in MF. Further well-designed clinical studies are needed for evaluating the significance of splenomegaly in MF.

## Figures and Tables

**Table 1:** Gene mutations with splenomgaly in patients with Myelofibrosis (MF).

Gene Mutation	Patient Characteristics	Results Associated with Splenomegaly	Reference
JAK2-V617F Homozygous mutation	210 patients with PMF - JAK2 V617F wild type (*n* = 111) - JAK2 heterozygous type (*n* = 109) - JAK2 homozygous type (*n* = 84)	Patients with JAK2 V617F homozygous type → Larger splenomegaly, higher WBC count than those with wild/heterogenous type (*p* < 0.001)	[38]
CALR mutation	617 patients with PMF. - JAK2 V617F mutation (*n* = 399) - CALR mutation (*n* = 140) - MPL mutation (*n* = 25) - triple negative (*n* = 53)	Patients with CALR mutation → longer large-splenomegaly-free survivals than remained patients (*p* < 0.001)	[39]
High risk mutations - AZXL1, EZH1, IDH1/2	85 MF patients treated with ruxolitinib - no mutation in AZXL1, EZH1 or IDH1/2 (*n* = 68) - ≥1 mutation in AZXL1, EZH1 or IDH1/2 (*n* = 27)	Patients with ≥ 1 mutation in AZXL1, EZH1 or IDH1/2 or those with ≥ 3 mutations of any types → significantly less likely have the response than those with no AZXL1, EZH1 or IDH1/2 (*p* = 0.01) or those with ≤ 2 mutation (*p* = 0.001)	[40]

**Table 2:** Janus kinase (JAK) inhibitors, as efficacious agents for splenomegaly in myelofibrosis.

Drug	Targets	Trial	Patients	Spleen Reduction Responses	References
Ruxotinib	JAK2/1/3 TYK2	COMFORT-I (randomized phase 3)	Int-2 & high risk MF	SVR ≥ 35% at 24 weeks - 41.9% in ruxolitinib group (vs. 0.7% in BAT group)	[42]
		COMFORT-II (randomized phase 3)	Int-2 & high risk MF	SVR ≥ 35% at 48 weeks - 28% in ruxolitinib group (vs. 0% in BAT group)	[43]
Pacritinib	JAK2/1/3 FLT3	PERSIST-I (randomized phase 3)	Int-1, Int-2 & high risk MF	SVR ≥ 35% at 24 weeks - 19% in pacritinib group (vs. 5% in placebo)	[45]
		PERSIST-II (randomized phase 3)	Int-1, Int-2 & high risk MF	SVR ≥ 35% at 24 weeks - 18% in pacritinib group (vs. 3% in placebo)	[46]
Momelotinib	JAK2/1/3 JNK2 CDK2	SIMPLIFY-I (randomized phase 3)	Int-1, Int-2 & high risk MF	SVR ≥ 35% at 24 weeks - 26.9% in momelotinib group (vs. 29% ruxolitinib)	[47]
		SIMPLIFY-II (randomized phase 3)	Int-1, Int-2 & high risk MF	SVR ≥ 35% at 24 weeks - momelotinib, not superior to placebo/ruxolitinib group	[48]
Fedratinib	JAK2/1/3 TYK2 FLT3 RET	JAKARTA (randomized phase 3)	Int-2 & high risk MF	SVR ≥ 35% at 24 weeks - 36% in 400 mg fedratinib group - 40% in 500 mg fedratinib group vs. 1% in placebo group	[50]
